# Medical quality assessment of tertiary public hospitals in Guangxi based on the national performance appraisal for tertiary public hospitals

**DOI:** 10.1097/MD.0000000000045390

**Published:** 2025-10-24

**Authors:** Lan Yang, Qiwei Chen, Liusi Wei, Lu Yang, Jun-Qiang Chen

**Affiliations:** a Department of Hospital Quality Management, The First Affiliated Hospital of Guangxi Medical University, Nanning, Guangxi, China; b School of Information and Management, Guangxi Medical University, Nanning, Guangxi, China; c Department of Education and Science, Maternal and Child Health Hospital of Guangxi Zhuang Autonomous Region, Nanning, Guangxi, China; d Department of Gastroenterology, Nanfang Hospital, Southern Medical University, Guangzhou, Guang Dong, China.

**Keywords:** entropy weight method (EWM), grey relational analysis (GRA), medical quality, national performance appraisal for tertiary public hospitals, technique for order preference by similarity to ideal solution (TOPSIS)

## Abstract

Tertiary public hospitals are the backbone of China’s healthcare system, yet their medical quality varies across regions. Since 2019, the national performance appraisal for tertiary public hospitals (NPA-TPH) has emphasized medical quality as the core evaluation dimension. However, comprehensive, objective, and dynamic assessment models remain limited, particularly in western regions such as Guangxi. This study evaluated medical quality in 23 tertiary general public hospitals in Guangxi from 2018 to 2021 using a hybrid model integrating entropy weight method (EWM), grey relational analysis (GRA), and technique for order preference by similarity to ideal solution (TOPSIS). Indicator weights were determined via EWM, performance association was assessed through GRA, and composite rankings were generated using TOPSIS. Kernel density estimation (KDE) was applied to visualize temporal changes in quality distribution. Overall medical quality showed an upward trend during the study period. Top-ranking hospitals consistently demonstrated strong performance, while others exhibited slow improvement or decline, indicating widening inter-hospital disparities. KDE results revealed a shift in score distribution from unimodal to multimodal patterns, suggesting structural differentiation and increasing polarization, characterized by “the strong getting stronger.” The EWM-GRA-TOPSIS model effectively provides an objective and comprehensive framework for medical quality evaluation. Findings indicate that while NPA-TPH policy has promoted quality improvement, disparities among tertiary public hospitals in Guangxi have intensified. Targeted, differentiated strategies are needed to enhance weaker hospitals and promote balanced regional healthcare development.

## 1. Introduction

Tertiary public hospitals represent the top-tier backbone of China’s public healthcare service system, symbolizing the highest level of regional medical technology and resource allocation. Under the national promotion of the tiered diagnosis and treatment policy, these institutions are tasked with increasing responsibilities, including receiving referrals and leading regional medical alliances. These expanded roles have significantly intensified the complexity of hospital governance and resource burdens.^[1–3]^ According to the *China Health Statistics Yearbook* (2023), there were approximately 1760 tertiary public hospitals across the country, accounting for over 1 billion outpatient visits and more than 100 million inpatient admissions annually – representing over 40% of the nation’s healthcare resources.^[4]^ The high concentration of medical resources and the inherent risk associated with intensive medical activities make tertiary hospitals particularly vulnerable to challenges in quality and safety management. In 2019, the General Office of the State Council issued the Guidelines on Strengthening Performance Appraisal in Tertiary Public Hospitals, formally initiating a national performance evaluation framework for these institutions. This assessment system encompasses 4 key dimensions: medical quality, operational efficiency, sustainable development, and satisfaction evaluation. Among these, medical quality was assigned the highest weighting, underscoring the central role it plays in the government’s strategy to enhance hospital governance and healthcare delivery at the tertiary level.^[5]^

Although the current performance appraisal system has yielded notable improvements in hospital operational efficiency and quality governance, several key challenges persist in the practical evaluation of medical quality. These challenges can be summarized in 3 aspects: First, the indicator system is complex and multidimensional, with considerable heterogeneity and inconsistent measurement scales across certain metrics. This makes it difficult to achieve effective integration using single-dimensional or subjectively weighted approaches. Second, data quality varies significantly across hospitals, and there is a lack of objective, data-driven models capable of minimizing human-induced bias in the evaluation process. Third, existing frameworks fall short in capturing the dynamic evolution of medical quality over time, limiting the ability to detect shifts in quality distribution throughout policy implementation phases.^[6–8]^

Therefore, this study focuses on 23 tertiary general public hospitals in the Guangxi Zhuang Autonomous Region. Based on the medical quality indicators derived from the national performance appraisal for tertiary public hospitals (NPA-TPH), this study integrates self-reported data collected over 4 consecutive years (2018–2021) to develop a comprehensive, three-stage hybrid evaluation model. The proposed model combines the entropy weight method (EWM), grey relational analysis (GRA), and the technique for order preference by similarity to ideal solution (TOPSIS) to enable objective weighting and comprehensive performance ranking. This model enables objective weighting and composite ranking to quantitatively assess overall performance in medical quality. In addition, we innovatively introduce the kernel density estimation (KDE) method to visualize the distributional characteristics and temporal dynamics of hospital quality scores, with the aim of identifying convergence or divergence trends in quality variation among institutions. Based on this framework, the study seeks to address the following core research questions: Under the national performance appraisal system for tertiary public hospitals, how can a scientific and objective model for evaluating medical quality be constructed? What was the overall status of medical quality among tertiary public hospitals in Guangxi from 2018 to 2021? Does the distribution of medical quality across hospitals exhibit signs of convergence, divergence, or polarization? By addressing these questions, the study aims to provide both a methodological reference and empirical evidence for evaluating medical quality, particularly in central and western regions of China. Moreover, the findings are intended to support local governments in optimizing performance appraisal mechanisms and promoting high-quality development of tertiary public hospitals.

## 2. Materials and methods

### 2.1. Study design

This study is grounded in the policy context of NPA-TPH, which has been implemented in China since 2019. The research focuses on 23 tertiary general public hospitals in the Guangxi Zhuang Autonomous Region and aims to conduct a comprehensive evaluation of medical quality. The data span from 2018 to 2021, covering critical periods before and after the policy implementation, with the goal of dynamically assessing trends in the quality of medical care across institutions. All data were obtained from the Reporting System of NPA-TPH in Guangxi, part of the Guangxi Health Comprehensive Management Sub-platform. Ethical approval was not required for this study.

To achieve a multidimensional and objective assessment, we constructed an integrated evaluation framework based on EWM-GRA-TOPSIS. Specifically, the EWM was applied to assign weights to each indicator based on information entropy, ensuring objectivity in weight distribution. GRA was then used to measure the degree of association between each hospital’s performance and an ideal reference sequence. Finally, the TOPSIS method was employed to calculate the relative closeness of each hospital to the ideal and negative ideal solutions, enabling composite ranking of medical quality across different years. In this study, the gray relational closeness coefficient was considered as the overall score for evaluating medical quality.

To further explore the evolutionary characteristics of medical quality distribution among tertiary public hospitals in Guangxi, we introduced the KDE method. KDE was used to smooth the quality scores and visualize their distribution, allowing us to identify annual trends and structural shifts in overall hospital quality levels.

### 2.2. Inclusion and exclusion criteria

Inclusion criteria were as follows: Nationally accredited tertiary general public hospitals located within the Guangxi Zhuang Autonomous Region; Hospitals that continuously participated in the NPA-TPH since its initiation in 2019, with complete self-reported data covering the period from 2018 to 2021; and Data submissions that passed quality control audits conducted by relevant departments of the Guangxi Health Commission.

Exclusion criteria included the following: Hospitals that experienced changes in accreditation level or were involved in institutional mergers during the study period; Hospitals with severe data incompleteness, defined as missing more than 15% of required values; Military hospitals, prison-affiliated hospitals, and other institutions operating under independent administrative systems with limited data transparency, which were excluded due to inaccessible or non-standardized reporting mechanisms; and Non-general hospitals, such as oncology hospitals and maternity hospitals, were excluded, as the study focused specifically on general tertiary hospitals.

### 2.3. Data source and preprocessing

The data used in this study were obtained from the NPA-TPH Reporting System embedded within the Comprehensive Management Sub-platform of the Guangxi Health Commission, specifically designed for tertiary public hospitals. To ensure comparability and standardized attribute treatment across indicators, the following data preprocessing steps were performed:

(1) Indicator selection: the qualitative indicator “Maintenance and Quality Control of Large Medical Equipment” was excluded to enhance horizontal comparability; The indicator “Single-Disease Quality Control” was removed due to high rates of missing values and inconsistent disease category inclusion across hospitals. As Guangxi only began implementing the national volume-based drug procurement policy in January 2020, the indicator “Proportion of Centralized Procurement Drugs Used” was excluded due to its incompatibility with 2018 to 2019 data. The indicator “Coverage Rate of Quality Nursing Service Wards” was consistently 100% across all hospitals from 2018 to 2021, resulting in zero information entropy and, consequently, a weight of zero. Based on the principle of entropy weighting, this indicator lacked discriminatory power and statistical significance, and was therefore excluded from the final model.
(2) Indicator transformation: the ordinal indicator “Level of Electronic Medical Record Application” was converted into numerical scores (Level 4 = 40, Level 6 = 60) to enable integration with other continuous variables.
(3) Indicator direction unification: reverse indicators (X_9_, X_10_, X_14_, X_16_, and X_21_) were transformed using reciprocal conversion to ensure that higher values consistently indicated better medical quality.
(4) Indicator normalization: All included indicators were rescaled to a [0, 1] range using min-max normalization, thereby eliminating the influence of differing units and magnitudes. To clarify the composition of the final evaluation system, Table 1 lists the 22 medical quality indicators used in the analysis, including their names, units, attributes, and corresponding codes.

**Table 1:** Overview of medical quality evaluation indicators.

Indicator	Unit	Indicator nature	Indicator code
Outpatient-to-inpatient discharge ratio	%	Positive	X_1_
Referral-out patient volume	Persons	Positive	X_2_
Day-surgery rate for elective procedures	%	Positive	X_3_
Surgical discharge ratio	%	Positive	X_4_
Minimally invasive surgery ratio	%	Positive	X_5_
Level-IV surgery ratio	%	Positive	X_6_
High-end medical service ratio	%	Positive	X_7_
High-end medical service revenue ratio	%	Positive	X_8_
Complication rate among surgical patients	%	Negative	X_9_
Surgical site infection rate in class I incisions	%	Negative	X_10_
Positive detection rate of large-scale medical equipment	%	Positive	X_11_
Participation rate in national external quality assessment (EQA) programs	%	Positive	X_12_
Pass rate in national external quality assessment (EQA) programs	%	Positive	X_13_
Mortality rate among low-risk patient groups	%	Negative	X_14_
Proportion of reviewed prescriptions among total prescriptions	%	Positive	X_15_
Intensity of antimicrobial drug use (DDD per 100 patient-days)	DDD	Negative	X_16_
Proportion of essential medicines in outpatient prescriptions	%	Positive	X_17_
Use rate of essential medicines among inpatients	%	Positive	X_18_
Proportion of essential drug varieties in total procurement	%	Positive	X_19_
Average appointment-based consultation rate among outpatients	%	Positive	X_20_
Average waiting time after appointment for outpatient services	min	Negative	X_21_
Level of electronic medical record (EMR) functionality	Grade	Positive	X_22_

### 2.4. Model selection

#### 2.4.1. Entropy weight method

The EWM, originally proposed by the American mathematician Claude Shannon in 1948, is a widely used objective weighting technique. Unlike subjective weighting approaches, the entropy method minimizes the influence of human bias by assigning weights based on the degree of variability inherent in each evaluation indicator. It evaluates the amount of information conveyed by each indicator through its dispersion across the dataset, without imposing assumptions on the underlying distribution.^[9–11]^ Due to its objectivity and robustness, the EWM has gained increasing application in multi-criteria decision-making (MCDM) in recent years. The computational procedure is outlined as follows:

Step1: Determine the weighting of the characteristics of the *j* indicator under the *j* characterization weight of the *j* indicator $P_{ij}$.

$$\begin{array}{l} P_{ij}=z_{ij}/\underset{i=1}{\overset{n}{\sum }}\;z_{ij} \end{array}$$ (1)

$$\begin{array}{l} z_{ij}>0and\underset{i=1}{\overset{n}{\sum }}\;z_{ij}>0 \end{array}$$

Step2: Find the entropy value of the *j* indicator $e_{j}$.

$$\begin{array}{l} \begin{array}{l} e_{j}=-k\underset{i=1}{\overset{m}{\sum }}\;p_{ij} \end{array} \end{array}$$ (2)

Eq: $k=\frac{1}{lnm}>0,$ $e_{j}>0$

Step3: Calculate the coefficient of variation for indicator *j* $h_{j}$

$$\begin{array}{l} \begin{array}{l} h_{j}=1-e_{j} \end{array} \end{array}$$ (3)

Step 4: Calculate the weighting coefficient of the indicator $w_{j}$

$$\begin{array}{l} \begin{array}{l} w_{j}=h_{j}/\underset{j=1}{\overset{n}{\sum }}\;h_{j},j-12n \end{array} \end{array}$$ (4)

#### 2.4.2. GRA-TOPSIS model

The TOPSIS, first introduced by Hwang and Yoon in 1981, is a widely adopted MCDM method for ranking and comparing multi-dimensional alternatives.^[12]^ Also referred to as the distance-to-ideal-solution method or the two-reference-point method, TOPSIS imposes minimal assumptions on the data, offers computational flexibility, and systematically evaluates the deviation of each alternative from the ideal solution. This enables an objective and transparent decision-making basis that accurately reflects the underlying characteristics of the problem. Despite its strengths, the classical TOPSIS approach is inherently limited to representing positional relationships, as it measures the relative performance of alternatives using Euclidean distance. Consequently, it is incapable of adequately distinguishing variation trends across alternatives or capturing the dynamic evolution of data sequences.^[13]^ To overcome these limitations, this study incorporates GRA, a method developed by Deng Julong, which quantifies the degree of association between sequences based on the geometric similarity of their data trajectories.^[14]^ GRA offers a more intuitive representation of the closeness between a comparative sequence and a reference sequence, thereby enhancing the interpretability of fuzzy interrelations and temporal trends within the evaluation objects.^[15]^ By integrating GRA with TOPSIS, both the positive and negative ideal distances and the gray relational coefficients can be standardized, allowing for the refinement of the relative closeness measure and improving the model’s capacity to reflect both static and dynamic relationships among alternatives. The steps for constructing the GRA-TOPSIS model are as follows:

**Step1:** Due to the difference in the unit of different evaluated indicators, it can be standardized:

for positive indicators:

$$\begin{array}{l} \begin{array}{l} z_{ij}=\frac{x_{ij}-\underset{1\leq i\leq m}{min}\;(x_{ij})}{\underset{1\leq i\leq m}{max}\;(x_{ij})-\underset{1\leq i\leq m}{min}\;(x_{ij})} \end{array} \end{array}$$ (5)

for negative indicators:

$$\begin{array}{l} \begin{array}{l} z_{ij}=\frac{\underset{1\leq i\leq m}{max}\;(x_{ij})-x_{ij}}{\underset{1\leq i\leq m}{max}\;(x_{ij})-\underset{1\leq i\leq m}{min}\;(x_{ij})} \end{array} \end{array}$$ (6)

From this, a decision matrix for normalizing the data can be obtained: $Z_{m\times n}$ = ${(z_{ij})}_{m\times n}$.

**Step 2:** Calculate the positive and negative ideal solutions of the weighted evaluated matrix. For the weighted evaluation matrix $Z=(Z_{ij}{)}_{m\times n}$, determine the positive ideal solution $Z^{+}=\left\{Z_{1}^{+}{,Z}_{2}^{+}{,,Z}_{j}^{+}{,,Z}_{n}^{+}\right\}$ and the negative ideal solution $Z^{\text{-}}=\left\{Z_{1}^{\text{-}}{,Z}_{2}^{\text{-}}{,,Z}_{j}^{\text{-}}{,,Z}_{n}^{\text{-}}\right\}$, then:

$$\begin{array}{l} \begin{array}{l} Z_{j}^{+}=\left\{\underset{1\leq i\leq m}{max}\;Z_{ij}|j=P,\underset{1\leq i\leq m}{min}\;Z_{ij}|j=N\right\} \end{array} \end{array}$$ (7)

$$\begin{array}{l} \begin{array}{l} Z_{j}^{-}=\left\{\underset{1\leq i\leq m}{min}\;Z_{ij}|j=P,\underset{1\leq i\leq m}{max}\;Z_{ij}|j=N\right\} \end{array} \end{array}$$ (8)

$Z_{j}^{+}$ represents the positive ideal solution for the j-th indicator, while $Z_{j}^{\text{-}}$ denotes the negative ideal solution for the j-th indicator. *P* refers to the positive indicators, and N refers to the negative indicators

**Step 3:** Calculate the Euclidean distances of the schemes to the positive and negative ideal solutions.

$$\begin{array}{l} \begin{array}{l} d_{i}^{+}=\sqrt{\overset{n}{\underset{j=1}{\sum }}{\left(Z_{ij}-Z_{j}^{+}\right)}^{2}},i=1,2,,m \end{array} \end{array}$$ (9)

$$\begin{array}{l} \begin{array}{l} d_{i}^{-}=\sqrt{\overset{n}{\underset{j=1}{\sum }}{\left(Z_{ij}-Z_{j}^{-}\right)}^{2}},i=1,2,,m \end{array} \end{array}$$ (10)

**Step 4:** Calculate the gray correlation between each scheme and the positive and negative ideal solutions. First, the absolute difference between the indicator data of each evaluation program and the positive and negative ideal solutions is calculated according to the weighted evaluation matrix, and the formula is:

$$\begin{array}{l} \begin{array}{l} \Delta_{ij}^{+}=\left|y_{ij}-y_{j}^{+}\right| \end{array} \end{array}$$ (11)

$$\begin{array}{l} \begin{array}{l} \Delta_{ij}^{-}=\left|y_{ij}-y_{j}^{-}\right| \end{array} \end{array}$$ (12)

**Step 5:** Calculate the gray correlation coefficient with positive and negative ideal solutions:

$$\begin{array}{l} \begin{array}{l} \xi_{ij}^{+}=\frac{\left(\underset{i}{min}\;\underset{j}{min}\;\Delta_{ij}^{+}+\rho \underset{i}{max}\;\underset{j}{max}\;\Delta_{ij}^{+}\right)}{\left(\Delta_{ij}^{+}+\rho \underset{i}{max}\;\underset{j}{max}\;\Delta_{ij}^{+}\right)} \end{array} \end{array}$$ (13)

$$\begin{array}{l} \begin{array}{l} \xi_{ij}^{-}=\frac{\left(\underset{i}{min}\;\underset{j}{min}\;\Delta_{ij}^{-}+\rho \underset{i}{max}\;\underset{j}{max}\;\Delta_{ij}^{-}\right)}{\left(\Delta_{ij}^{-}+\rho \underset{i}{max}\;\underset{j}{max}\;\Delta_{ij}^{-}\right)} \end{array} \end{array}$$ (14)

ρ: distinguishing coefficient,0< ρ <1, typically set to 0.5.

**Step 6:** Calculate the gray correlation between each scenario and the positive and negative ideal solutions:

$$\begin{array}{l} \begin{array}{l} r_{i}^{+}=\frac{1}{m}\sum_{j=1}^{n}\xi_{ij}^{+} \end{array} \end{array}$$ (15)

$$\begin{array}{l} \begin{array}{l} r_{i}^{-}=\frac{1}{m}\sum_{j=1}^{n}\xi_{ij}^{-} \end{array} \end{array}$$ (16)

**Step 7:** Normalization is applied separately to the Euclidean distance and gray relational degree:

$$\begin{array}{l} \begin{array}{l} \begin{array}{ll} D_{i}^{+}=\frac{d_{i}^{+}}{\underset{1\leq i\leq m}{max}\;d_{i}^{+}}, & D_{i}^{-}=\frac{d_{i}^{-}}{\underset{1\leq i\leq m}{max}\;d_{i}^{-}} \end{array} \end{array} \end{array}$$ (17)

$$\begin{array}{l} \begin{array}{l} \begin{array}{ll} R_{i}^{+}=\frac{r_{i}^{+}}{\underset{1\leq i\leq m}{max}\;r_{i}^{+}}, & R_{i}^{-}=\frac{r_{i}^{-}}{\underset{1\leq i\leq m}{max}\;r_{i}^{-}} \end{array} \end{array} \end{array}$$ (18)

**Step 8:** Calculate the comprehensive distance of each evaluation scheme to the positive and negative ideal solutions. The combined formula for normalized Euclidean distance and gray relational degree is given as:

$$\begin{array}{l} \begin{array}{l} H_{i}^{+}=\alpha R_{i}^{+}+\beta D_{i}^{-} \end{array} \end{array}$$ (19)

$$\begin{array}{l} \begin{array}{l} H_{i}^{-}=\alpha R_{i}^{-}+\beta D_{i}^{+} \end{array} \end{array}$$ (20)

0≤αβ≤1 and α+β= 1, α and β represent the preference weights for shape and distance, respectively. Both are set to 0.5 in this study. The comprehensive distances of each evaluation scheme to the positive and negative ideal solutions are denoted as $H_{i}^{+}$ and $H_{i}^{\text{\textasciitilde{}}\text{-}}$.

**Step 9:** Calculate the relative closeness.

$$\begin{array}{l} \begin{array}{l} L_{i}=\frac{H_{i}^{+}}{H_{i}^{+}+H_{i}^{-}} \end{array} \end{array}$$ (21)

#### 2.4.3. Kernel density estimation

Kernel density estimation (KDE) is an important non-parametric statistical technique used to smoothly estimate the probability density function of a continuous variable without assuming a predefined distribution form. In this study, KDE is employed to visualize the dynamic evolutionary trends in medical quality across the sampled hospitals.^[16,17]^ The formula for Kernel Density Estimation is given as:

$$\begin{array}{l} \begin{array}{l} f(x)=\frac{1}{nh}\underset{i=1}{\overset{n}{\sum }}\;k\left(\frac{x-X_{i}}{h}\right) \end{array} \end{array}$$ (22)

In this study, K(.) is the Kernel function, where $X_{1},,X_{n}$ are the evaluation scores of sample hospitals based on the GRA-TOPSIS calculation, x is the mean, n is the number of observations, and h is the bandwidth. The Gaussian kernel function, known for its high accuracy, is used to analyze the dynamic distribution characteristics of sample hospitals’ medical quality during the observation period.

## 3. Results

### 3.1. Descriptive statistical analysis of indicator

As shown in Table 2, most positive indicators (e.g., X_2_, X_11_, X_12_, X_13_, X_15_, X_17_, and X_20_) remained relatively stable or showed gradual improvement over the study period, reflecting the continuous enhancement of medical quality among tertiary public hospitals in Guangxi. Indicator X_1_ experienced a temporary decline in 2020 due to the impact of the COVID-19 pandemic in China but rebounded in 2021 to 15.44, nearly returning to its 2018 level. Some negative indicators, such as X_9_ and X_10_, remained at low levels throughout the study period, indicating effective control in areas such as infection prevention and rational drug use. However, indicators X_14_ and X_21_ demonstrated an upward trend, suggesting ongoing challenges in medical quality and efficiency in Guangxi’s tertiary public hospitals. Notably, indicator X_16_, which reflects the intensity of antimicrobial use, showed a steady decline from 43.13 DDDs in 2018 to 37.91 DDDs in 2021, aligning with national antimicrobial stewardship policies and indicating improved antibiotic management.

**Table 2:** Descriptive statistics of 22 medical service quality indicators from 2018–2021.

Indicator	2018		2019		2020		2021	
	Mean ± SD	Median	Mean ± SD	Median	Mean ± SD	Median	Mean ± SD	Median
X_1_	15.53 ± 4.15	14.47	15.27 ± 4.48	14.04	14.12 ± 4.05	12.51	15.44 ± 4.55	14.40
X_2_	1132 ± 2829	115	2700 ± 5690	560	2439 ± 4205	725	3582 ± 6508	1085
X_3_	5.28 ± 6.83	2.38	7.38 ± 6.68	6.11	8.77 ± 6.71	9.01	11.19 ± 8.37	8.85
X_4_	29.85 ± 7.98	28.06	28.70 ± 5.21	28.07	29.92 ± 5.15	29.15	29.53 ± 4.82	29.69
X_5_	18.93 ± 8.67	16.44	19.76 ± 7.81	18.39	18.49 ± 5.94	17.29	18.00 ± 4.56	17.89
X_6_	14.20 ± 6.10	14.46	15.87 ± 5.63	15.80	17.23 ± 4.97	16.66	18.00 ± 4.67	17.91
X_7_	0.01 ± 0.05	0.00	0.01 ± 0.02	0.00	0.07 ± 0.22	0.00	0.16 ± 0.35	0.00
X_8_	0.00	0.00	0.01 ± 0.02	0.00	0.03 ± 0.06	0.00	0.14 ± 0.47	0.00
X_9_	0.54 ± 0.51	0.47	0.91 ± 1.06	0.64	0.90 ± 0.39	0.91	0.76 ± 0.33	0.76
X_10_	0.28 ± 0.24	0.19	0.19 ± 0.19	0.14	0.13 ± 0.20	0.08	0.13 ± 0.22	0.06
X_11_	86.17 ± 6.13	86.61	88.50 ± 3.44	88.11	86.86 ± 4.32	87.08	89.71 ± 3.48	89.31
X_12_	61.78 ± 38.35	81.60	82.06 ± 19.56	90.40	90.58 ± 11.65	96.02	91.15 ± 10.05	93.09
X_13_	74.81 ± 40.59	96.18	95.17 ± 3.84	95.63	96.53 ± 2.99	96.79	97.45 ± 2.01	98.00
X_14_	0.02 ± 0.03	0.00	0.01 ± 0.01	0.00	0.02 ± 0.02	0.01	0.02 ± 0.03	0.01
X_15_	8.81 ± 6.84	6.89	7.16 ± 4.77	6.89	8.85 ± 6.30	7.94	9.88 ± 9.25	8.48
X_16_	43.13 ± 10.09	39.82	43.16 ± 8.64	41.02	40.08 ± 6.50	39.35	37.91 ± 4.59	37.91
X_17_	59.91 ± 17.60	62.94	66.20 ± 9.01	66.41	66.66 ± 6.71	66.10	67.97 ± 5.77	67.97
X_18_	97.48 ± 3.35	98.89	97.56 ± 2.51	98.21	97.14 ± 2.59	97.89	97.03 ± 1.87	97.56
X_19_	42.30 ± 7.98	40.95	41.06 ± 3.72	41.11	40.74 ± 4.09	40.08	40.55 ± 3.66	39.64
X_20_	43.91 ± 20.52	48.51	47.06 ± 23.29	50.51	52.93 ± 26.23	58.69	57.34 ± 21.54	62.87
X_21_	11.83 ± 14.56	5.57	14.58 ± 13.06	14.65	15.59 ± 9.54	13.37	17.60 ± 6.63	16.59
X_22_	21.30 ± 13.59	30.00	29.57 ± 10.65	30.00	37.83 ± 4.22	40.00	40.00 ± 4.26	40.00

### 3.2. Weight distribution of medical quality evaluation indicators for tertiary public hospitals in Guangxi

The entropy weights for the medical quality indicators across sample hospitals from 2018 to 2021 are presented in Table 3. Using 2021 as an example, the top 5 indicators by entropy weight were: X_8_ (0.261), X_7_ (0.203), X_2_ (0.125), X_15_ (0.047), and X_5_ (0.017). These results suggest that, under the current policy environment and operational context of public hospitals, indicators reflecting hospital functional positioning, service capacity, rational drug use, and technical capability contribute more significantly to the differentiation of medical quality. In contrast, certain structural or IT-related indicators such as X_12_ (0.013), X_13_ (0.016) and X_20_ (0.017) received relatively low entropy weights. This indicates limited variation in these metrics across hospitals and time periods, thus reducing their discriminatory power in quality assessment. Overall, the EWM effectively assigned weights based on the intrinsic variability of objective data, minimizing subjective bias and providing a robust foundation for the subsequent GRA-TOPSIS composite evaluation model.

**Table 3:** Entropy weights of medical quality evaluation indicators from 2018–2021.

Indicator	2018	2019	2020	2021
X_1_	0.027	0.029	0.027	0.027
X_2_	0.129	0.126	0.116	0.125
X_3_	0.064	0.043	0.043	0.037
X_4_	0.027	0.017	0.032	0.038
X_5_	0.029	0.045	0.020	0.017
X_6_	0.015	0.021	0.022	0.022
X_7_	0.243	0.238	0.253	0.203
X_8_	0.257	0.294	0.226	0.261
X_9_	0.009	0.006	0.015	0.018
X_10_	0.015	0.008	0.008	0.007
X_11_	0.010	0.020	0.014	0.014
X_12_	0.025	0.011	0.016	0.013
X_13_	0.022	0.007	0.008	0.016
X_14_	0.006	0.008	0.010	0.006
X_15_	0.033	0.039	0.038	0.047
X_16_	0.008	0.013	0.015	0.017
X_17_	0.008	0.013	0.020	0.026
X_18_	0.007	0.008	0.008	0.013
X_19_	0.012	0.011	0.033	0.035
X_20_	0.019	0.021	0.027	0.017
X_21_	0.007	0.011	0.019	0.026
X_22_	0.028	0.011	0.032	0.016

### 3.3. Evaluation results of medical quality in tertiary public hospitals in Guangxi (2021)

In this study, we first used the 2021 dataset to perform initial evaluation and ranking. A standardized decision matrix of quality indicators was first constructed (Table S1, Supplemental Digital Content, https://links.lww.com/MD/Q433). Based on this matrix, the gray relational coefficients between each hospital and the ideal reference sequence were calculated (Tables S2 and S3, Supplemental Digital Content, https://links.lww.com/MD/Q433). By integrating the entropy-derived indicator weights (Table 3), each hospital’s superiority score, inferiority score, and gray relational closeness coefficient were obtained. The final evaluation results are summarized in Table 4. As shown, Hospital H2 achieved the highest closeness score (0.600), ranking first, indicating the strongest overall performance across all evaluated indicators. Hospitals H8 (0.516) and H19 (0.483) ranked second and third, respectively. In contrast, Hospitals H7 (0.340), H21 (0.344) and H11 (0.347) received the lowest scores, ranking 23rd, 22nd and 21st respectively, reflecting relatively weaker performance in overall medical quality. Overall, substantial differences in closeness scores were observed among the 23 tertiary public hospitals in Guangxi, highlighting the uneven distribution of medical quality across the region. While some hospitals demonstrated strength in service capacity and quality management, others still exhibited considerable gaps, suggesting the need for further improvements in performance management and internal quality enhancement.

**Table 4:** GRA-TOPSIS closeness results for sample hospitals in 2021.

Hospital	Superiority scores	Inferiority scores	Grey relational closeness	Ranking
H1	0.903	0.893	0.447	4
H2	0.900	0.881	0.600	1
H3	0.862	0.909	0.423	5
H4	0.845	0.948	0.353	17
H5	0.852	0.937	0.360	13
H6	0.847	0.947	0.359	14
H7	0.834	0.961	0.340	23
H8	0.892	0.885	0.516	2
H9	0.841	0.953	0.347	20
H10	0.857	0.929	0.375	8
H11	0.841	0.953	0.347	21
H12	0.856	0.937	0.364	12
H13	0.849	0.936	0.365	11
H14	0.842	0.953	0.357	15
H15	0.859	0.935	0.373	9
H16	0.849	0.932	0.371	10
H17	0.865	0.928	0.376	7
H18	0.848	0.942	0.357	16
H19	0.877	0.912	0.483	3
H20	0.858	0.931	0.409	6
H21	0.837	0.955	0.344	22
H22	0.843	0.952	0.350	19
H23	0.845	0.948	0.352	18

GRA = grey relational analysis, TOPSIS = technique for order preference by similarity to ideal solution.

### 3.4. Evaluation results of medical quality in tertiary public hospitals in Guangxi from 2018 to 2021

Using the same steps, medical quality scores and rankings for tertiary public hospitals in Guangxi from 2018 to 2020 were also calculated. The aggregated results for the four-year period are presented in Table 5. Hospitals H1, H3, H8, and H19 consistently ranked within the top 5, despite minor fluctuations in their annual scores, indicating a stable and high level of medical quality. Hospital H2 demonstrated notable progress, with its score increasing to 0.600 in 2021, placing it at the top of the ranking-a substantial improvement from 2018, reflecting significant advancements in quality management. Hospital H16 exhibited a continuous upward trend after 2020, reaching 9th place in 2021, which may be attributed to its successful re-accreditation as a Grade III-A institution that year. In contrast, Hospital H7 experienced a steady decline in performance, falling to 23rd place in 2021 – the lowest among all hospitals-warranting urgent attention. Hospital H20 showed considerable year-to-year fluctuations in ranking, suggesting potential instability in its quality management practices. Hospitals H22 and H23 consistently remained at the bottom of the evaluation over the four-year period, with 2021 scores of 0.350 and 0.352, respectively, indicating relatively weak service capacity and quality management. These hospitals should be prioritized in future improvement initiatives. Overall, the findings reveal structural disparities in medical quality among tertiary general hospitals in Guangxi. Based on the evaluation outcomes, it is recommended that targeted, differentiated quality improvement strategies be developed to promote sustained enhancement of regional healthcare services.

**Table 5:** GRA-TOPSIS closeness results for sample hospitals from 2018–2021.

Hospital	2018		2019		2020		2021	
	Scores	Ranking	Scores	Ranking	Scores	Ranking	Scores	Ranking
H1	0.465	3	0.419	3	0.429	4	0.447	4
H2	0.372	10	0.354	8	0.370	7	0.600	1
H3	0.538	1	0.611	1	0.488	3	0.423	5
H4	0.366	13	0.350	13	0.355	14	0.353	17
H5	0.373	9	0.350	12	0.358	13	0.360	13
H6	0.344	23	0.352	10	0.353	16	0.359	14
H7	0.346	20	0.347	15	0.337	23	0.340	23
H8	0.398	5	0.443	2	0.630	1	0.516	2
H9	0.360	14	0.340	21	0.347	19	0.347	20
H10	0.401	4	0.364	5	0.371	5	0.375	8
H11	0.357	18	0.349	14	0.350	17	0.347	21
H12	0.375	8	0.354	7	0.363	9	0.364	12
H13	0.346	22	0.342	20	0.362	10	0.365	11
H14	0.358	16	0.345	17	0.347	18	0.357	15
H15	0.376	7	0.342	18	0.359	12	0.373	9
H16	0.346	21	0.331	23	0.369	8	0.371	10
H17	0.383	6	0.350	11	0.370	6	0.376	7
H18	0.372	11	0.346	16	0.361	11	0.357	16
H19	0.368	12	0.361	6	0.506	2	0.483	3
H20	0.359	15	0.394	4	0.346	20	0.409	6
H21	0.351	19	0.353	9	0.354	15	0.344	22
H22	0.357	17	0.337	22	0.343	22	0.350	19
H23	0.526	2	0.342	19	0.344	21	0.352	18

GRA = grey relational analysis, TOPSIS = technique for order preference by similarity to ideal solution.

### 3.5. Kernel density estimation of medical quality scores

**Figure 1.**
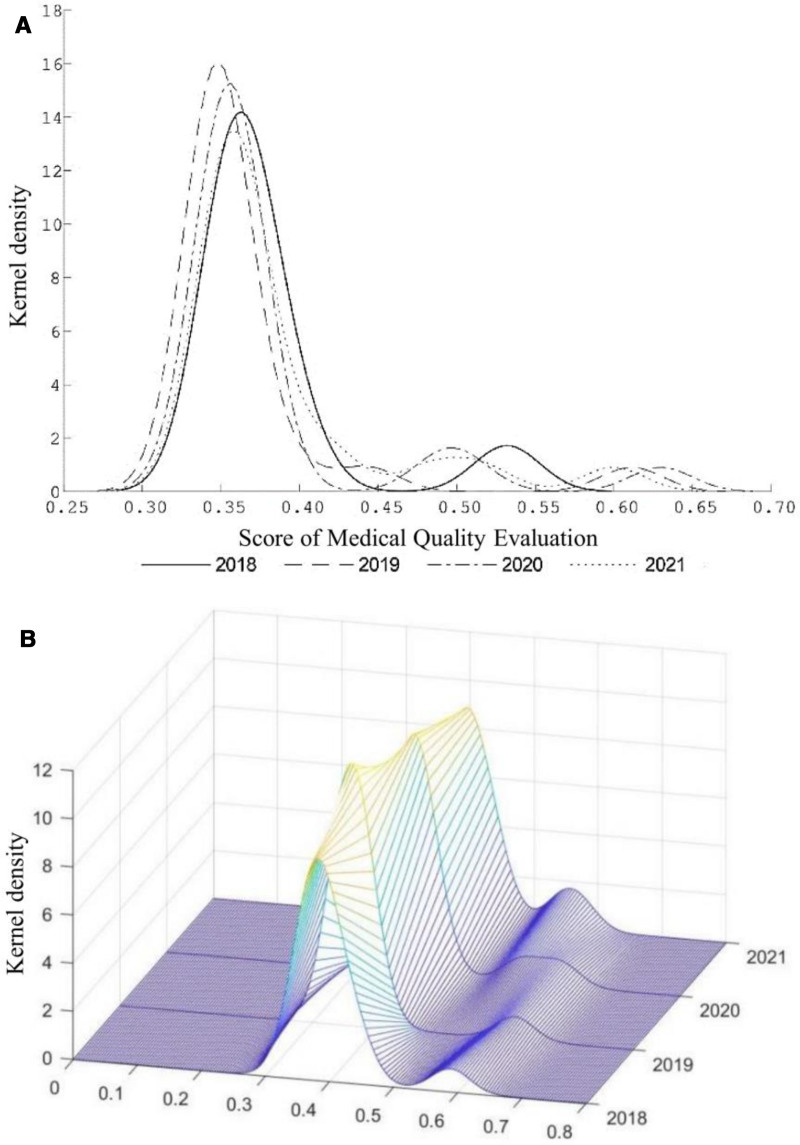
Dynamic evolution trend of medical quality in tertiary general public hospitals in Guangxi from 2018–2021. (A) Two-dimensional kernel density estimation of medical quality. (B) Three-dimensional kernel density estimation of medical quality.

Both Two-dimensional and three-dimensional KDE plots were generated to visualize score distributions, as shown in Figure 1. In terms of distributional shifts, the density curves exhibited a “left-right” movement over time. In 2018 and 2019, the peak density was concentrated around 0.35. In 2020, the distribution shifted rightward, and by 2021, the overall distribution further moved toward 0.45, indicating a fluctuating upward trend in medical quality performance under the influence of NPA-TPH policy. Regarding peak height and shape, the 2018 curve displayed a high, narrow peak, reflecting concentrated performance levels among hospitals. In contrast, the peaks in 2020 and 2021 became lower and flatter, suggesting a widening gap in quality performance across hospitals. The increased distribution width over time further implies greater dispersion and heterogeneity in hospital medical quality. From the perspective of modality, the distribution in 2018 exhibited a clear bimodal structure, indicating a degree of polarization in hospital performance. By 2020, the emergence of multiple peaks pointed to an expanding performance gap, potentially driven by differences in management capacity and COVID-19 prevention and control policy. In summary, the KDE results reveal that while the overall medical quality of tertiary hospitals in Guangxi has improved under the NNPA-TPH policy, inter-hospital disparities have become increasingly pronounced. These findings highlight the need for regionally coordinated quality management and differentiated policy interventions to address structural imbalances.

## 4. Discussion

This study focused on 23 tertiary general public hospitals in the Guangxi Zhuang Autonomous Region, drawing on data from the reporting platfrom of NPA-TPH covering the period from 2018 to 2021. A comprehensive evaluation model integrating EWM, GRA and TOPSIS was constructed, and the KDE method was applied to systematically assess the medical quality levels and their dynamic distribution trends from both cross-sectional and longitudinal perspectives. The main findings indicate that the medical quality of tertiary public hospitals in Guangxi showed a steady upward trend during the study period, reflecting the guiding effect of the NNPA-TPH policy on quality improvement. Inter-hospital comparisons revealed significant disparities, with some hospitals consistently maintaining leading performance while others showed slower progress and widening gaps. KDE analysis further demonstrated that the distribution of medical quality evolved from a unimodal to a multimodal pattern, indicating structural differentiation among hospitals and a growing trend of polarization, characterized by “the strong getting stronger.”

The results of this study align with existing domestic and international research in terms of both model applicability and the observed trends in quality evolution, while also demonstrating a degree of extensibility and regional specificity. First, regarding the scientific validity and feasibility of the model, previous literature has widely applied MCDM methods in the evaluation of medical quality.^[18,19]^ Chang et al employed a combined TOPSIS and RSR approach to comprehensively rank the medical quality of tertiary traditional Chinese medicine hospitals in China, confirming the effectiveness of such models in handling high-dimensional indicator systems.^[20]^ Wang et al integrated fuzzy AHP with TOPSIS to develop an evaluation framework for ward system maturity, emphasizing the flexibility and practical value of these methods in hospital performance assessment.^[21]^ These studies provide both a methodological foundation and conceptual innovation for the model of EWM-GRA-TOPSIS in this research.

Second, in terms of performance improvement trends and regional differentiation among hospitals, the findings of this study are also consistent with previous empirical research. Ye et al conducted an empirical analysis of tertiary public hospitals in Sichuan Province and found that while hospital service capacity improved overall under the impetus of performance-related policies, significant intra-regional disparities persisted.^[22]^ Zhao et al in a study focusing on the Guangzhou area, further revealed that medical service quality exhibited dynamic evolution and stratification across different subregions, highlighting the need for dynamic analytical tools to better understand structural changes in quality.^[23]^ In this study, KDE was introduced to characterize the trajectory of medical quality evolution from a distributional perspective, revealing a clear trend of structural divergence, marked by “the strong getting stronger” and widening disparities. This trend largely reflects the underlying differences among tertiary public hospitals in terms of functional positioning, governance capacity, and data execution capabilities. Although the sampled hospitals share the same accreditation level, they vary significantly in terms of regional hierarchy, service orientation, and institutional roles. Provincial or autonomous region level hospitals typically demonstrate stronger resource allocation capabilities, more robust technical platforms and disciplinary systems, and higher responsiveness to performance assessments, enabling a more effective feedback mechanism for continuous improvement.^[24]^ In contrast, many municipal-level hospitals, while possessing basic management frameworks, remain relatively weak in terms of quality governance infrastructure, data management capabilities, and performance-incentive alignment, making it difficult to translate assessment pressure into quality improvement.^[25–27]^ Moreover, mismatches between assessment indicators and hospital disciplinary structures also contribute to performance disadvantages: hospitals with weaker specialty development or imbalanced structures may struggle to meet technical benchmarks, leading to structural misalignment in evaluation outcomes. These factors collectively contribute to the observed non-uniform improvements and structural differentiation in medical quality across tertiary hospitals.^[28]^ Supporting this, Li et al noted in their study of secondary public hospitals in Guangzhou that public hospitals across different levels and administrative hierarchies face significant differences in resource integration, discipline development, and governance capacity, which in turn influence the transmission and translation of performance policies.^[29]^ This aligns closely with the findings of the present study, where heterogeneous improvement trajectories were observed even among tertiary hospitals, underscoring the region and institution-specific nature of policy implementation outcomes.

Despite the methodological rigor applied in model construction and data analysis, this study has several limitations. First, the sample is limited to 23 tertiary general hospitals in Guangxi, restricting the generalizability of the findings to a broader national context. Second, some indicators are derived from administrative reporting systems, which may vary in terms of standardization and objectivity, potentially affecting data reliability. In addition, although the indicator system covers multiple dimensions, it does not include “soft” quality indicators such as patient experience or staff satisfaction, limiting the comprehensiveness of quality measurement. Future research should expand the sample to include hospitals from eastern, central, and western China across different development levels to improve horizontal comparability. Moreover, incorporating subjective quality dimensions and applying structural equation modeling may help uncover the behavioral mechanisms behind quality improvement. Spatial analysis tools could also be introduced to model patterns of regional clustering and diffusion in hospital quality performance.

## 5. Conclusion

Medical quality in tertiary public hospitals in Guangxi has shown an overall upward trend. However, inter-hospital disparities have widened, and the distribution of quality has shifted from concentration toward differentiation, exhibiting a structural pattern of “the strong getting stronger.” This study offers a valuable evidence base and quantitative support for optimizing performance assessment policies and enhancing regional healthcare governance, as well as for improving quality management in tertiary public hospitals.

## Acknowledgments

The authors thank Scott S. Tighe, professor from Western Oregon University, professor Zhenyu, Ma, from Guangxi Medical University, Chief physician Dunke Jiang, Head nurse Fanglan Wu from the First Affiliated Hospital of Guangxi Medical University for their assistances and suggestions in the preparation and editing of this manuscript.

## Author contributions

**Conceptualization:** Lan Yang, Jun-Qiang Chen.

**Formal analysis:** Lan Yang, Lu Yang.

**Methodology:** Lu Yang.

**Software:** Qiwei Chen, Liusi Wei.

**Supervision:** Jun-Qiang Chen.

**Validation:** Qiwei Chen.

**Visualization:** Lan Yang.

**Writing – original draft:** Lan Yang, Qiwei Chen.

**Writing – review & editing:** Lan Yang, Qiwei Chen, Liusi Wei.

## Supplementary Material


