# Supplementary material for: Medical quality assessment of tertiary public hospitals in Guangxi based on the national performance appraisal for tertiary public hospitals
Source: Medicine (Baltimore). 2025 Oct 24;104(43):e45390. doi: 10.1097/MD.0000000000045390 (PMC12558211; doi:10.1097/MD.0000000000045390)
Supplement: Supplementary file 1 [file medi-104-e45390-s001.docx]

TableS1 Decision Matrix of 2021 Medical Quality Indicators for Sample Hospitals

| **Hospital/Indicator** | X1 | X2 | X3 | X4 | X5 | X6 | **......** | **......** | X7 | X21 | X22 |
| --- | --- | --- | --- | --- | --- | --- | --- | --- | --- | --- | --- |
| H1 | 0.019 | 0.125 | 0.037 | 0.038 | 0.008 | 0.022 | **......** | **......** | 0.000 | 0.016 | 0.008 |
| H2 | 0.020 | 0.009 | 0.029 | 0.029 | 0.005 | 0.014 | **......** | **......** | 0.128 | 0.003 | 0.016 |
| H3 | 0.010 | 0.015 | 0.002 | 0.006 | 0.003 | 0.011 | **......** | **......** | 0.069 | 0.013 | 0.008 |
| H4 | 0.014 | 0.004 | 0.012 | 0.016 | 0.006 | 0.012 | **......** | **......** | 0.000 | 0.014 | 0.008 |
| H5 | 0.007 | 0.014 | 0.012 | 0.017 | 0.007 | 0.012 | **......** | **......** | 0.000 | 0.016 | 0.008 |
| H6 | 0.000 | 0.002 | 0.011 | 0.001 | 0.000 | 0.012 | **......** | **......** | 0.000 | 0.015 | 0.000 |
| H7 | 0.006 | 0.000 | 0.004 | 0.002 | 0.007 | 0.017 | **......** | **......** | 0.000 | 0.004 | 0.008 |
| H8 | 0.013 | 0.031 | 0.021 | 0.013 | 0.009 | 0.018 | **......** | **......** | 0.203 | 0.011 | 0.008 |
| H9 | 0.004 | 0.006 | 0.003 | 0.000 | 0.005 | 0.015 | **......** | **......** | 0.000 | 0.004 | 0.008 |
| H10 | 0.008 | 0.038 | 0.020 | 0.005 | 0.008 | 0.014 | **......** | **......** | 0.000 | 0.008 | 0.016 |
| H11 | 0.007 | 0.001 | 0.000 | 0.013 | 0.004 | 0.006 | **......** | **......** | 0.000 | 0.000 | 0.008 |
| H12 | 0.005 | 0.002 | 0.033 | 0.012 | 0.003 | 0.010 | **......** | **......** | 0.000 | 0.015 | 0.008 |
| H13 | 0.019 | 0.014 | 0.012 | 0.014 | 0.003 | 0.003 | **......** | **......** | 0.020 | 0.015 | 0.008 |
| H14 | 0.027 | 0.003 | 0.029 | 0.002 | 0.004 | 0.000 | **......** | **......** | 0.000 | 0.015 | 0.000 |
| H15 | 0.008 | 0.000 | 0.007 | 0.004 | 0.007 | 0.011 | **......** | **......** | 0.000 | 0.017 | 0.008 |
| H16 | 0.011 | 0.011 | 0.018 | 0.006 | 0.006 | 0.004 | **......** | **......** | 0.023 | 0.013 | 0.008 |
| H17 | 0.003 | 0.009 | 0.037 | 0.012 | 0.017 | 0.008 | **......** | **......** | 0.000 | 0.001 | 0.008 |
| H18 | 0.006 | 0.013 | 0.011 | 0.012 | 0.006 | 0.008 | **......** | **......** | 0.000 | 0.005 | 0.008 |
| H19 | 0.006 | 0.003 | 0.013 | 0.021 | 0.005 | 0.004 | **......** | **......** | 0.191 | 0.026 | 0.008 |
| H20 | 0.009 | 0.101 | 0.013 | 0.011 | 0.006 | 0.005 | **......** | **......** | 0.000 | 0.025 | 0.008 |
| H21 | 0.003 | 0.003 | 0.004 | 0.008 | 0.007 | 0.007 | **......** | **......** | 0.000 | 0.011 | 0.008 |
| H22 | 0.016 | 0.002 | 0.005 | 0.007 | 0.005 | 0.005 | **......** | **......** | 0.000 | 0.014 | 0.008 |
| H23 | 0.009 | 0.002 | 0.011 | 0.012 | 0.005 | 0.006 | **......** | **......** | 0.000 | 0.006 | 0.008 |

Table S2 2021 Grey Relational Coefficients between Medical Quality Indicators and Optimal Reference Sequence

| **Hospital/Indicator** | ***X*_1_** | ***X*_2_** | ***X*_3_** | ***X*_4_** | ***X*_5_** | ***X*_6_** | **......** | **......** | ***X*_7_** | ***X*_21_** | ***X*_22_** |
| --- | --- | --- | --- | --- | --- | --- | --- | --- | --- | --- | --- |
| H1 | 0.939 | 1.000 | 1.000 | 1.000 | 0.931 | 1.000 | **......** | **......** | 0.971 | 0.930 | 0.944 |
| H2 | 0.946 | 0.531 | 0.939 | 0.935 | 0.914 | 0.948 | **......** | **......** | 0.967 | 0.848 | 1.000 |
| H3 | 0.886 | 0.542 | 0.785 | 0.799 | 0.903 | 0.924 | **......** | **......** | 1.000 | 0.905 | 0.944 |
| H4 | 0.907 | 0.520 | 0.836 | 0.852 | 0.922 | 0.930 | **......** | **......** | 0.943 | 0.914 | 0.944 |
| H5 | 0.863 | 0.540 | 0.835 | 0.857 | 0.923 | 0.934 | **......** | **......** | 0.999 | 0.928 | 0.944 |
| H6 | 0.828 | 0.515 | 0.831 | 0.779 | 0.883 | 0.930 | **......** | **......** | 0.885 | 0.923 | 0.894 |
| H7 | 0.861 | 0.512 | 0.796 | 0.781 | 0.925 | 0.965 | **......** | **......** | 0.897 | 0.855 | 0.944 |
| H8 | 0.899 | 0.582 | 0.887 | 0.835 | 0.941 | 0.972 | **......** | **......** | 0.950 | 0.895 | 0.944 |
| H9 | 0.847 | 0.523 | 0.790 | 0.774 | 0.911 | 0.954 | **......** | **......** | 0.990 | 0.854 | 0.944 |
| H10 | 0.874 | 0.600 | 0.883 | 0.799 | 0.934 | 0.946 | **......** | **......** | 0.960 | 0.875 | 1.000 |
| H11 | 0.867 | 0.514 | 0.778 | 0.837 | 0.906 | 0.896 | **......** | **......** | 0.984 | 0.833 | 0.944 |
| H12 | 0.852 | 0.515 | 0.966 | 0.830 | 0.901 | 0.916 | **......** | **......** | 0.964 | 0.922 | 0.944 |
| H13 | 0.940 | 0.541 | 0.839 | 0.842 | 0.903 | 0.874 | **......** | **......** | 0.979 | 0.919 | 0.944 |
| H14 | 1.000 | 0.517 | 0.937 | 0.785 | 0.908 | 0.859 | **......** | **......** | 0.934 | 0.918 | 0.894 |
| H15 | 0.873 | 0.512 | 0.812 | 0.792 | 0.929 | 0.923 | **......** | **......** | 0.929 | 0.937 | 0.944 |
| H16 | 0.887 | 0.533 | 0.871 | 0.801 | 0.920 | 0.883 | **......** | **......** | 0.961 | 0.906 | 0.944 |
| H17 | 0.844 | 0.530 | 0.994 | 0.831 | 1.000 | 0.905 | **......** | **......** | 0.975 | 0.839 | 0.944 |
| H18 | 0.859 | 0.538 | 0.833 | 0.832 | 0.923 | 0.905 | **......** | **......** | 0.951 | 0.862 | 0.944 |
| H19 | 0.861 | 0.518 | 0.845 | 0.886 | 0.917 | 0.881 | **......** | **......** | 0.949 | 1.000 | 0.944 |
| H20 | 0.877 | 0.847 | 0.845 | 0.826 | 0.920 | 0.889 | **......** | **......** | 0.943 | 0.991 | 0.944 |
| H21 | 0.845 | 0.518 | 0.796 | 0.812 | 0.927 | 0.899 | **......** | **......** | 0.902 | 0.893 | 0.944 |
| H22 | 0.919 | 0.515 | 0.801 | 0.807 | 0.915 | 0.888 | **......** | **......** | 0.975 | 0.916 | 0.944 |
| H23 | 0.875 | 0.516 | 0.831 | 0.830 | 0.915 | 0.894 | **......** | **......** | 0.907 | 0.868 | 0.944 |

Table S3 2021 Grey Relational Coefficients between Medical Quality Indicators and Negative-Ideal Reference Sequence

| **Hospital/Indicator** | ***X*_1_** | ***X*_2_** | ***X*_3_** | ***X*_4_** | ***X*_5_** | ***X*_6_** | **......** | **......** | ***X*_7_** | ***X*_21_** | ***X*_22_** |
| --- | --- | --- | --- | --- | --- | --- | --- | --- | --- | --- | --- |
| H1 | 0.875 | 0.512 | 0.778 | 0.774 | 0.945 | 0.859 | **......** | **......** | 0.909 | 0.889 | 0.944 |
| H2 | 0.869 | 0.934 | 0.819 | 0.817 | 0.964 | 0.901 | **......** | **......** | 0.913 | 0.979 | 0.894 |
| H3 | 0.927 | 0.901 | 0.989 | 0.960 | 0.976 | 0.924 | **......** | **......** | 0.885 | 0.913 | 0.944 |
| H4 | 0.904 | 0.971 | 0.918 | 0.893 | 0.954 | 0.919 | **......** | **......** | 0.935 | 0.904 | 0.944 |
| H5 | 0.953 | 0.907 | 0.920 | 0.888 | 0.953 | 0.914 | **......** | **......** | 0.885 | 0.891 | 0.944 |
| H6 | 1.000 | 0.989 | 0.924 | 0.991 | 1.000 | 0.918 | **......** | **......** | 1.000 | 0.895 | 1.000 |
| H7 | 0.956 | 0.999 | 0.972 | 0.988 | 0.951 | 0.886 | **......** | **......** | 0.984 | 0.970 | 0.944 |
| H8 | 0.912 | 0.810 | 0.863 | 0.913 | 0.935 | 0.880 | **......** | **......** | 0.928 | 0.924 | 0.944 |
| H9 | 0.973 | 0.961 | 0.982 | 1.000 | 0.967 | 0.896 | **......** | **......** | 0.892 | 0.971 | 0.944 |
| H10 | 0.940 | 0.778 | 0.867 | 0.961 | 0.942 | 0.903 | **......** | **......** | 0.918 | 0.946 | 0.894 |
| H11 | 0.948 | 0.994 | 1.000 | 0.911 | 0.972 | 0.954 | **......** | **......** | 0.897 | 1.000 | 0.944 |
| H12 | 0.966 | 0.988 | 0.800 | 0.919 | 0.978 | 0.932 | **......** | **......** | 0.915 | 0.896 | 0.944 |
| H13 | 0.874 | 0.904 | 0.914 | 0.905 | 0.976 | 0.981 | **......** | **......** | 0.902 | 0.899 | 0.944 |
| H14 | 0.828 | 0.982 | 0.821 | 0.982 | 0.971 | 1.000 | **......** | **......** | 0.944 | 0.900 | 1.000 |
| H15 | 0.941 | 1.000 | 0.949 | 0.971 | 0.947 | 0.925 | **......** | **......** | 0.949 | 0.882 | 0.944 |
| H16 | 0.925 | 0.927 | 0.879 | 0.958 | 0.957 | 0.969 | **......** | **......** | 0.918 | 0.912 | 0.944 |
| H17 | 0.978 | 0.939 | 0.782 | 0.918 | 0.883 | 0.944 | **......** | **......** | 0.906 | 0.992 | 0.944 |
| H18 | 0.959 | 0.914 | 0.922 | 0.917 | 0.954 | 0.944 | **......** | **......** | 0.927 | 0.962 | 0.944 |
| H19 | 0.955 | 0.977 | 0.908 | 0.859 | 0.960 | 0.971 | **......** | **......** | 0.929 | 0.833 | 0.944 |
| H20 | 0.937 | 0.564 | 0.908 | 0.925 | 0.957 | 0.962 | **......** | **......** | 0.934 | 0.839 | 0.944 |
| H21 | 0.976 | 0.978 | 0.972 | 0.943 | 0.949 | 0.951 | **......** | **......** | 0.979 | 0.926 | 0.944 |
| H22 | 0.893 | 0.988 | 0.964 | 0.949 | 0.962 | 0.963 | **......** | **......** | 0.905 | 0.902 | 0.944 |
| H23 | 0.938 | 0.986 | 0.924 | 0.919 | 0.962 | 0.956 | **......** | **......** | 0.973 | 0.954 | 0.944 |
